# NetCrafter: ontology-derived gene network modeling and interpretation

**DOI:** 10.1093/bib/bbaf631.024

**Published:** 2025-12-12

**Authors:** Yeji Lee, Sukjoon Yoon

**Affiliations:** Department of Biological Sciences, Sookmyung Women’s University, Seoul, Republic of Korea; Department of Biological Sciences, Sookmyung Women’s University, Seoul, Republic of Korea

## Abstract

Understanding the complex nature of multi-functional interactions among genes is crucial for interpreting omics data. We developed NetCrafter, an ontology-driven platform for constructing de novo gene networks that are specific to each input gene list and quantitatively defined by ontology-weighted similarity. By incorporating the probabilistic association of ontology or curated gene sets into a weighted Tanimoto similarity metric, NetCrafter transforms enrichment results into quantitative semantic similarity scores between genes, enabling the creation of context-specific statistical networks. These networks can be further decomposed into optimal sub-networks, facilitating multi-functional interpretation and the identification of gene interaction hotspots. NetCrafter also supports meta-analysis across heterogeneous datasets by integrating multiple gene lists through consensus ontology scoring. Importantly, this list-specific, quantitative framework reveals functional hotspots and target-biomarker relationships – even in cases where ontology terms alone are not predictive of node-level attributes such as CRISPR efficacy. NetCrafter provides an interactive platform for constructing and interpreting dynamic, context-specific gene networks, leveraging ontology-based functional associations to uncover underlying mechanisms and identify key nodes. It is freely available at https://netcrafter.sookmyung.ac.kr and integrated into Q-omics platform (https://qomics.io) to enhance the utility of cancer omics data.

